# Dr. James Wylie Bowden

**Published:** 1910-06

**Authors:** 


### OBITUARY

Dr. James Wylie Bowden, of Yonkers, N. Y., died at his home in that city May 3, 1910, of apoplexy, aged 62 years. He was graduated in medicine from Bellevue Hospital Medical College in 1869 and during the succeeding sixteen years was clinical assistant to Dr. James R. Wood, of whom he was a warm admirer. Soon afterward he removed to Yonkers, retired from active practice, and devoted his attention mainly to life insurance, being medical director of one of the large New York companies. A man of a religious turn, he was deeply interested in the work of Saint John's Episcopal Church at Yonkers. Besides, he possessed an attractive personality, and was greatly respected by a large community.

